# Hypercalcemia and Renal Mass: A Diagnostically Challenging Case

**DOI:** 10.7759/cureus.16718

**Published:** 2021-07-29

**Authors:** Sunita Karki, Sneha Galiveeti, Vivien Leung

**Affiliations:** 1 Internal Medicine, BronxCare Health System, Bronx, USA; 2 Endocrinology, Diabetes and Metabolism, BronxCare Health System, Bronx, USA

**Keywords:** hypercalcemia, renal cancer, vitamin-d, pthrp, paraneoplastic syndrome

## Abstract

Hypercalcemia of malignancy frequently occurs in patients with solid tumors as a paraneoplastic syndrome known as humoral hypercalcemia of malignancy (HHM), caused by the secretion of parathyroid hormone-related peptide (PTHrP). On the other hand, 1,25-dihydroxyvitamin D [1,25(OH)2D]-mediated hypercalcemia is a less common cause of hypercalcemia of malignancy and is mostly observed in lymphoma patients. Here, we report an interesting case of a 77-year-old male nursing home resident with suspected renal cell carcinoma (RCC) presenting with severe hypercalcemia (18.7 mg/dL), which was initially presumed to be HHM. However, workup revealed nonsuppressed parathyroid hormone, low PTHrP, and elevated 25-hydroxyvitamin D and 1,25(OH)2D levels. Steroids were initiated due to an inadequate response to bisphosphonate therapy and elevated vitamin D metabolites, resulting in further reduction in serum calcium levels. This case highlights the need to consider multiple concurrent etiologies in the differential diagnosis of severe hypercalcemia, including the possible role of calcitriol-mediated hypercalcemia in RCC.

## Introduction

Primary hyperparathyroidism and malignancy, consisting of nearly 90% of the cases, are the most common causes of hypercalcemia. Other significant causes of hypercalcemia include familial hypocalciuric hypercalcemia, tertiary hyperparathyroidism, hyperthyroidism, drug effects, sarcoidosis, tuberculosis, and immobilization. Malignancy results in acute extreme and symptomatic hypercalcemia, necessitating hospitalization. Humoral hypercalcemia of malignancy (HHM) due to excess production of parathyroid hormone-related peptide (PTHrP) is the most common subtype in solid tumors. Excessive synthesis of 1,25-dihydroxyvitamin D [1,25(OH)2D] is a far less common form of paraneoplastic syndrome, which is usually reported in patients with lymphomas [1,2]. Here, we report a case of severe hypercalcemia in an elderly patient with a renal tumor who presented with unusual laboratory findings of normal parathyroid hormone (PTH), low PTHrP, and elevated 1,25(OH)2D levels.

## Case presentation

A 77-year-old male nursing home resident presented with hypercalcemia. He had various medical conditions including type 2 diabetes, hypertension, peripheral artery disease, a history of above-the-knee amputation, coronary artery disease, and a renal mass suspicious for renal cell carcinoma (RCC) that was discovered during a prior hospitalization.

The patient was noted to have suffered a progressive decline in mental status in the nursing home for the past two weeks. The initial serum calcium levels were 13 mg/dL. Intravenous fluids were initiated at the nursing home. Repeat laboratory testing over one week showed worsening hypercalcemia, with serum calcium levels of 16.8 mg/dL, and the patient was sent to the emergency room for further evaluation.

In the emergency room, he was found to be mildly tachycardic (heart rate of 101 beats per minute), and his other vital signs were within the normal range. Physical examination revealed dry mucous membranes and decreased responsiveness to painful stimuli. Laboratory findings revealed elevated serum calcium levels of 18 mg/dL (corrected for albumin: 18.7 mg/dL) and acute kidney injury with serum creatinine levels of 2.4 mg/dL (baseline creatinine: 0.8 mg/dL) three months prior to the presentation.

The patient’s medications included losartan-hydrochlorothiazide, insulin glargine, carvedilol, and aspirin. A review of his nursing home records revealed a prescription of 2,000 IU cholecalciferol daily three months prior for 25-hydroxyvitamin D [25(OH)D] deficiency (17.0 ng/mL) which was discontinued after the initial increase in serum calcium levels. His calcium levels had last been measured three months prior to admission to the emergency room and were normal (9 mg/dL).

Further investigations revealed normal PTH levels of 36.5 pg/mL (reference range: 10-65 pg/mL), low PTHrP levels of 10 pg/mL (reference range: 14-27 pg/mL), elevated 25(OH)D levels of 200 ng/mL (reference range: 30-50 ng/mL), and elevated 1,25(OH)2D levels of 86 pg/mL (reference range: 18 -72 pg/mL).

**Figure 1:**
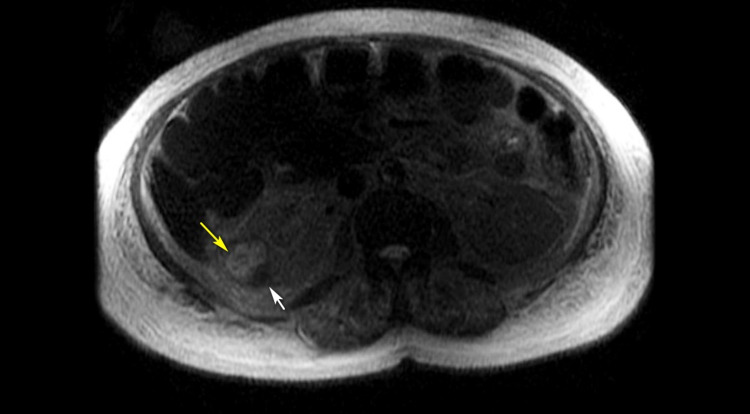
MRI of the abdomen and pelvis. A 3-cm heterogeneous signal lesion exophytic from the lateral portion of the mid-right kidney was observed (yellow arrow). A cystic lesion was found adjacent to this lesion and slightly inferior, suggestive of a hemorrhagic cyst (white arrow). MRI: magnetic resonance imaging

**Figure 2:**
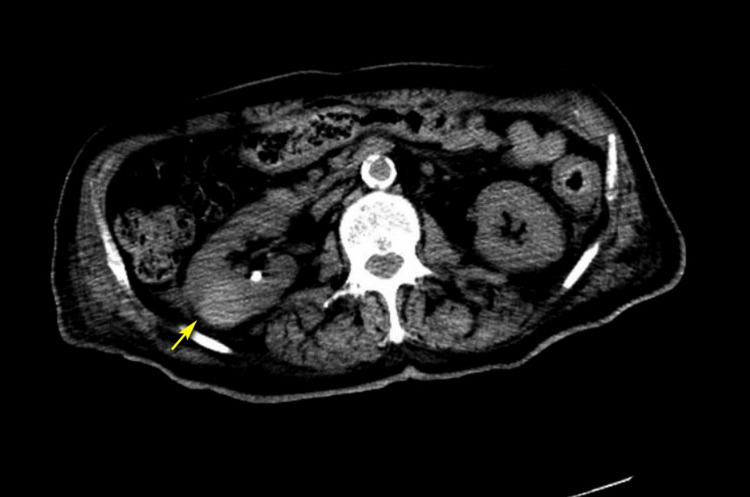
CT scan of the abdomen and pelvis. A hyperattenuating lesion measuring 3.1 × 3 cm was noted in the maximal axial dimension, which was suspicious for a renal mass (yellow arrow). CT: computed tomography

**Figure 3:**
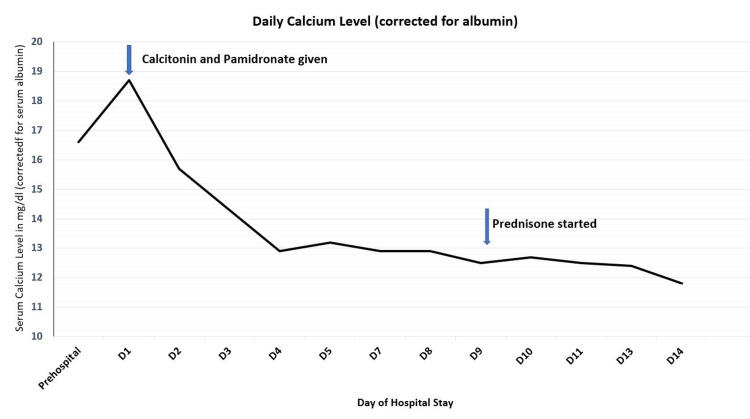
Daily calcium levels (corrected for albumin).

Thyroid function test, angiotensin-converting enzyme levels, and serum and urine protein electrophoresis showed normal results. A whole-body bone scan was unremarkable, but a CT scan of the abdomen and pelvis revealed a hyperattenuating lesion of 3.1 × 3.0 cm deforming the contour of the right kidney, with a similar finding on MRI (Figures 1, 2). The patient was treated with intravenous hydration, calcitonin, and pamidronate, and his calcium levels decreased to 12 mg/dL by day three. Prednisone was initiated due to an incomplete response to pamidronate and elevated levels of 25(OH)D and 1,25-(OH)2D resulting in a further reduction of serum calcium levels to 10.4 mg/dL (Figure 3 and Table 1).

**Table 1:** Laboratory findings at presentation and follow-up. PTH: parathyroid hormone; PTHrP: parathyroid hormone-related peptide

Laboratory findings	Day 1	Day 8 after initiating pamidronate and calcitonin	Day 14 after prednisone
Serum total calcium (8.5–10.5 mg/dL)	18.0	11.3	10.4
Serum ionized calcium (1.15–1.29 mmol/L)	2.49	-	-
Serum phosphorus (2.5–4.5 mg/dL)	4.8	3.4	6.4
Serum albumin (3.20–4.6 g/dL)	3.1	2.0	2.2
Serum blood urea nitrogen (8–26 mg/dL)	56.0	59.0	80.0
Serum creatinine (0.5–1.5 mg/dL)	2.4	2.5	6.0
Serum PTH (10–65 pg/mL)	36.5	-	11.9
Serum PTHrP (14–27 pg/mL)	10	-	-
Serum total vitamin D 25(OH) (ng/mL)	200	-	158.0
Serum 1,25 dihydroxyvitamin D (pg/mL)	86	-	-

However, the patient’s clinical condition remained poor, and his family decided not to pursue any further investigations, including a kidney biopsy. The patient was discharged to hospice care, where he died 20 days later.

## Discussion

Primary hyperparathyroidism and malignancy, comprising nearly 90% of the cases, are the most common causes of hypercalcemia. Other significant causes of hypercalcemia include familial hypocalciuric hypercalcemia, tertiary hyperparathyroidism, hyperthyroidism, drug effects, sarcoidosis, tuberculosis, and immobilization. Hypercalcemia of malignancy is the most common cause of hypercalcemia among hospitalized patients and can manifest suddenly and severely, with altered mental status, as seen in the present case [1,2]. It is important to consider all potential factors in the workup of patients with severe hypercalcemia and solid tumors as there may be concurrent etiologies or mechanisms contributing to their overall presentation.

Lung and kidney cancers are the most common malignancies that cause hypercalcemia. Humoral hypercalcemia in RCC was first described in 1941 by Albright who suggested that the tumor secretes a factor similar to PTH that is responsible for hypercalcemia. The factor was identified in the late 1980s and was named PTHrP. Hypercalcemia of malignancy causes a paraneoplastic syndrome known as HHM, in which tumor cells secrete PTHrP. The N-terminal sequence of PTHrP is similar to that of PTH, which allows it to bind to the PTH receptor. PTHrP and PTH act via G-protein-coupled receptors and share similar features. Both stimulate increased nephrogenic cyclic adenosine monophosphate and inhibit the reabsorption of phosphate in the proximal tubule. In addition, they both stimulate osteoclastic bone resorption. PTHrP differs from PTH in its limited ability to stimulate 1α-hydroxylase in the proximal tubule and calcium reabsorption in the distal tubule. Levels of 1,25(OH)2D are usually elevated in primary hyperparathyroidism and are generally suppressed in HHM. Suppression of 1,25(OH)2D in HHM may be a result of elevated serum calcium inhibiting the activity of renal 1α-hydroxylase. Another potential explanation is the release of a factor by the tumor that inhibits renal 1α-hydroxylase [2,3].

We were unable to attribute hypercalcemia to HHM in our patient due to the low levels of PTHrP (10 pg/mL; reference range: 14-27 pg/mL). However, hypercalcemia in RCC has been attributed to other non-PTHrP humoral factors, such as interleukin (IL)-6, IL-1, tumor necrosis factor-α, and transforming growth factors alpha and beta. Some studies have reported that IL-6 acts synergistically when expressed with PTHrP to activate osteoclasts resulting in bone resorption. Prostaglandins have also been implicated in the pathogenesis of hypercalcemia in patients with RCC by stimulating bone resorption [4]. However, IL-6 and prostaglandins were not measured in our patient due to a lack of available testing.

Vitamin D intoxication is an uncommon condition that was considered in the differential diagnosis of hypercalcemia in our patient due to his initial 25(OH)D levels of 200 ng/dL. However, our patient had been given a relatively low dose of 2,000 IU of cholecalciferol daily for three months. We did not suspect an unintentional overdose of vitamin D since he resided in a nursing home and his medications were supervised. In previously reported cases, vitamin D-induced hypercalcemia occurred after the administration of >100,000 IU (2,500 µg) per day for at least one month due to manufacturing and labeling errors of dietary supplements. Levels of 25(OH)D were reported to be 1,220 and 645 ng/dL in two cases [5]. In contrast, the cholecalciferol dose and 25(OH)D levels in our patient did not seem high enough to fully account for this degree of hypercalcemia.

Despite extreme hypercalcemia in our patient, his initial PTH levels were inappropriately normal (36.5 pg/mL; reference range: 10-65 pg/mL). Hypercalcemia due to malignancy typically presents with low or suppressed PTH levels due to negative feedback. Primary hyperparathyroidism is a common cause of inappropriate PTH elevation, which may coexist in patients with malignancy. Ectopic PTH production by tumor cells is rare but has been previously described in metastatic RCC [6,7]. However, the PTH levels in our patient did not rebound during his hospital course despite improved calcium levels making parathyroid autonomy a less likely scenario. A parathyroid scan to exclude the concurrent causes was not performed in our patient as the family opted not to pursue an aggressive workup.

Several medications can lead to hypercalcemia, such as calcium carbonate (present in calcium-containing antacids), lithium, a large dose of vitamin A, vitamin D supplements and its analogs, and thiazide diuretics. Our patient took hydrochlorothiazide, which may have contributed to hypercalcemia; however, mild hypercalcemia of 11 mg/dL is typical in patients who take thiazides and have concurrent conditions associated with hypercalcemia. Severe hypercalcemia is rarely associated with hydrochlorothiazide [7,8]. Therefore, thiazide use would be a less likely cause of hypercalcemia in our patient.

In our patient, elevated levels of 1,25(OH)2D, despite marked calcium, indicated inappropriate or ectopic calcitriol production, possibly via a renal tumor. Although 1,25(OH)2D-mediated hypercalcemia is classically described in lymphomas, a previous case study reported simultaneous elevation of PTHrP and 1,25(OH)2D in a patient with metastatic RCC and hypercalcemia that responded favorably to glucocorticoids [3]. In another study, circulating 1,25(OH)2D concentrations in patients with RCC-associated hypercalcemia were found to be rarely suppressed, suggesting the possibility of dysregulated calcitriol production in this cancer type [9]. Furthermore, 1,25(OH)2D-mediated hypercalcemia is usually responsive to glucocorticoid therapy via inhibition of 1α-hydroxylase, which converts 25(OH)D to its active form 1,25(OH)2D. Glucocorticoids were initiated as soon as we recognized the elevated levels of 1,25(OH)2D and suboptimal response to bisphosphonates, leading to further improvement in our patient’s calcium levels.​​​

## Conclusions

Our patient with a renal mass presented with severe hypercalcemia and unusual laboratory findings of normal PTH, low PTHrP, and elevated 25(OH)D and 1,25(OH)2D levels. Several mechanisms, including non-PTHrP humoral factors and dysregulated renal tumor-associated calcitriol production, could have been implicated, along with nonmalignancy factors such as vitamin D supplementation, parathyroid autonomy, and thiazide use. We suspect that abnormal 1,25(OH)2D production by the renal tumor may have played a significant role in the hypercalcemia seen in the patient, although the mechanisms are not well understood. While intravenous bisphosphonates and supportive measures are the first-line treatment in patients with established or suspected RCC presenting with severe hypercalcemia, it is imperative to check vitamin D metabolites as calcitriol-mediated mechanisms may be involved which preferentially respond to glucocorticoid therapy.
